# Left ventricular diastolic dysfunction: the missing window for early intervention in diabetic cardiomyopathy

**DOI:** 10.3389/fendo.2026.1921804

**Published:** 2026-07-30

**Authors:** Yuting Lin, Yebin Xia, Jinjian Guo

**Affiliations:** Department of Cardiology, The Second People’s Hospital Affiliated to Fujian University of Traditional Chinese Medicine, Fuzhou, Fujian, China

**Keywords:** cardio-metabolic disease, diabetic cardiomyopathy, heart failure with preserved ejection fraction, left ventricular diastolic dysfunction, type 2 diabetes mellitus

## Introduction

Type 2 diabetes mellitus (T2DM) is one of the leading contributors to cardiovascular morbidity and mortality worldwide. Even in the absence of hypertension or coronary artery disease, T2DM independently increases the risk of heart failure (1). This diabetes-associated myocardial disorder, commonly referred to as diabetic cardiomyopathy (DCM), is increasingly recognized as an independent clinical entity (2). However, its current clinical definition remains largely restricted to the advanced stage characterized by overt heart failure, particularly heart failure with preserved ejection fraction (HFpEF). This heart failure-centered paradigm may not fully capture an extended preclinical phase during which myocardial dysfunction is already present, but remains clinically silent. Among the early functional abnormalities, left ventricular diastolic dysfunction (LVDD) has been consistently reported as one of the earliest detectable manifestations of diabetic myocardial involvement, often preceding systolic dysfunction and symptomatic heart failure.

## Subsections relevant for the subject

LVDD is widely regarded as one of the earliest functional abnormalities in DCM. Prior to the development of overt systolic impairment (3), a substantial proportion of asymptomatic patients with T2DM already exhibit echocardiographic evidence of LVDD (4). These abnormalities are characterized by impaired myocardial relaxation, increased ventricular stiffness, and elevated filling pressures. A growing body of evidence suggests that these functional changes are driven by multiple interrelated pathophysiological processes, including myocardial lipotoxicity, mitochondrial dysfunction, oxidative stress, chronic low-grade inflammation, microvascular rarefaction, and interstitial fibrosis (5). Collectively, these mechanisms progressively impair ventricular compliance during the early stages of the disease, while the left ventricular ejection fraction remains preserved. These observations suggest that myocardial injury in diabetes may occur much earlier than clinically apparent heart failure, challenging current assumptions regarding the timing of DCM diagnosis.

We propose that the diagnostic paradigm of DCM should shift from the traditional heart failure-oriented model toward a framework centered on LVDD. In current clinical practice, DCM is most often recognized only after the development of symptomatic heart failure or overt structural remodeling. This approach inevitably leads to diagnosis at relatively advanced stages of the disease, when myocardial fibrosis and microvascular injury may already be irreversible. In contrast, LVDD represents a distinct transitional stage linking metabolic dysregulation to overt heart failure. Rather than being regarded as a secondary echocardiographic finding, LVDD should be considered as the first clinically detectable manifestation of diabetic myocardial injury. From this perspective, DCM may be better conceptualized as a continuous disease spectrum, within which LVDD represents the earliest clinically meaningful stage. This LVDD-centered framework shifts the diagnostic emphasis away from end-stage heart failure toward early functional impairment, thereby enabling the identification of high-risk individuals before irreversible myocardial remodeling occurs.

The close relationship between LVDD and HFpEF further supports the clinical relevance of this concept. A growing body of evidence suggests that DCM represents a major pathogenic contributor to the development of HFpEF (6). Many of the characteristic features of HFpEF, including myocardial fibrosis, endothelial dysfunction, chronic low-grade inflammation, and reduced ventricular compliance, are already present during the stage of LVDD in patients with diabetes (7). Accordingly, LVDD may represent a preclinical stage along the trajectory toward HFpEF, identifying a critical therapeutic window during which disease progression may still be modifiable. Failure to recognize and intervene at this stage may allow continued myocardial remodeling, ultimately leading to irreversible structural changes and progression to overt heart failure.

Despite its clinical relevance, systematic assessment of diastolic function remains underutilized in the routine evaluation of patients with T2DM. Conventional echocardiographic parameters, including the *E*/*e*′ ratio, left atrial volume index, and tricuspid regurgitation velocity, provide important information regarding left ventricular diastolic function (8). In addition, advanced imaging modalities such as speckle-tracking echocardiography, left atrial strain analysis, and cardiac magnetic resonance imaging have demonstrated greater sensitivity in detecting subclinical myocardial dysfunction (9). The integration of these techniques into routine screening strategies may facilitate the earlier identification of diabetic patients at increased cardiovascular risk.

## Discussion

**Figure 1:**
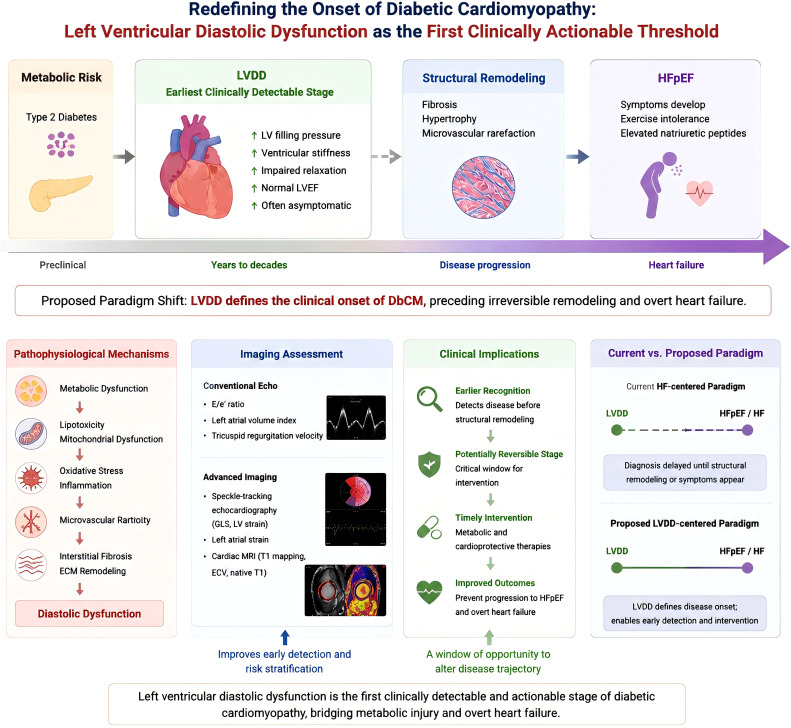
Left ventricular diastolic dysfunction as the earliest clinically actionable stage of diabetic cardiomyopathy.

In summary, LVDD may represent not only an early functional abnormality of DCM but also a potential mechanistic and clinical bridge between metabolic dysfunction and overt heart failure. We propose that the diagnostic paradigm of DCM could benefit from a shift away from a purely heart failure-centered model toward a framework that recognizes LVDD as an early clinically relevant stage of disease. Such a perspective may refine the current screening strategies and highlight an important therapeutic window during which disease progression may still be modifiable. However, this interpretation remains hypothesis-generating and requires further validation. The prognostic significance of LVDD in predicting progression to HFpEF is not fully established, and standardized diagnostic criteria in diabetic populations remain to be clarified. Future studies should focus on the validation of LVDD as a clinically meaningful diagnostic target and the exploration of whether early intervention at this stage can alter the disease trajectory and prevent progression to HFpEF and overt heart failure (**Figure 1**).
